# Predicting habitat suitability of the critically endangered Be'er Sheva fringe‐fingered lizard

**DOI:** 10.1002/ece3.70108

**Published:** 2024-08-16

**Authors:** Sefi J. A. Horesh, Ofer Ovadia

**Affiliations:** ^1^ Department of Life Sciences Ben‐Gurion University of the Negev Be'er Sheva Israel; ^2^ Midbarium Animal Park Be'er Sheva Israel; ^3^ The Goldman Sonnenfeldt School of Sustainability and Climate Change Ben‐Gurion University of the Negev Be'er Sheva Israel

**Keywords:** *Acanthodactylus beershebensis*, anthropogenic changes, area conservation, endemic species, habitat requirements, species distribution model

## Abstract

Anthropogenic changes, such as land use, are the main drivers causing climate change and biodiversity loss, with hundreds of thousands of species lacking sufficient habitats for their populations to persist and likely to go extinct within decades. Endemic species are more susceptible to habitat changes and are at the forefront of the biodiversity crisis. We used species distribution models to generate a relative habitat suitability map and identified the habitat requirements of the critically endangered and endemic Be'er Sheva fringe‐fingered lizard (*Acanthodactylus beershebensis*). The model showed that the species' suitable habitats are associated with arid loess plains characterized by scattered, low vegetation cover, primarily on north‐facing aspects, suggesting that these species‐specific habitat requirements limit its distribution. The size of the potentially suitable area within the species' historical range is 1350.73 km^2^. However, anthropogenic changes decreased the remaining suitable habitat to 995.04 km^2^. Most of this area is unprotected and at risk of further adverse anthropogenic effects. Only 91.72 km^2^ of this area is protected by the Israel Nature and Parks Authority, and 587.11 km^2^ may be considered indirectly protected because it is within military firing zones. Our study is the first attempt to map the remaining suitable habitat of *A. beershebensis* based on the results of a species distribution model. The results of this model can assist in prioritizing the protection of areas needed for the conservation of this critically endangered and endemic lizard species.

## Graphical abstract

We used species distribution models to map the distribution and habitat requirements of the endemic and critically endangered Be'er Sheva fringe‐fingered lizard (*Acanthodactylus beershebensis*). The size of the potentially suitable area within the species historical range is 1350.73 km^2^. However, anthropogenic changes shrunk the remaining suitable habitat to 995.04 km^2^. Only 91.72 km^2^ of this area is protected by the Israel Nature and Parks Authority, and 587.11 km^2^ may be considered indirectly protected because it is within military firing zones. Our study is the first attempt to map the remaining suitable habitat of the critically endangered lizard species *A. beershebensis* based on the results of a species distribution model.

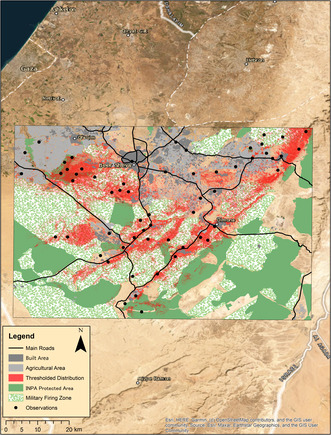

## 1 INTRODUCTION

Anthropogenic changes vastly accelerate climate change and biodiversity loss (Díaz et al., 2019; Parmesan & Yohe, 2003; Thomas et al., 2004), with extinction rates being exceptionally high (Ceballos et al., 2015). These processes significantly affect the stability of ecological systems while negatively altering ecosystem functions and services on which humanity strongly depends (Cardinale et al., 2012; Ceballos et al., 2015; Díaz et al., 2019). Indeed, Díaz et al. (2019) found that if human actions continue under “business as usual” scenarios, biodiversity and ecosystem services will sharply decline to alarming levels.

Globally, land‐use change is the most significant anthropogenic driver impacting ecosystems, leading to habitat loss and habitat deterioration (Díaz et al., 2019; IPBES, 2019). Human actions have reduced global terrestrial habitat integrity, resulting in hundreds of thousands of species that lack sufficient habitats for their populations to persist and are likely to go extinct within decades (Díaz et al., 2019; IPBES, 2019). Conservationists must gain vast knowledge regarding species' habitat requirements to assist in conserving natural ecosystems and mitigating biodiversity loss. This requires allocating considerable effort to study endangered species while aiming to relieve adverse pressures on their populations (Ceballos et al., 2015; Díaz et al., 2019).

The Be'er Sheva fringe‐fingered lizard, *Acanthodactylus beershebensis*, is a ground‐dwelling lizard endemic to the loess plains of the Negev Desert and southern Judea Desert of Israel (Bouskila & Amitai, 2003; Goldberg, 2014; Hawlena et al., 2010). Severe population declines reported in the late twentieth century due to habitat disturbance, destruction, and fragmentation associated with massive agricultural transformations have led this species to be classified as Critically Endangered by the IUCN (Bouskila, 2002; Carretero et al., 2011; Hawlena et al., 2010; Werner & Disi, 2006). When developing conservation plans for such endemic species, one must consider that they are typically more susceptible to habitat changes and thus experience increased population declines (Díaz et al., 2019; IPBES, 2019), leading to high extinction rates (Işik, 2011). The latter is often further magnified owing to their narrow geographical range, specialized ecological niches, and small population sizes (Işik, 2011).

A central challenge in conservation biology is understanding the main drivers of species distribution in general and endangered species; in particular, for further ecological and conservation goals (Petford et al., 2019). Doing so provides the basic knowledge required for planning effective conservation strategies (Guisan & Thuiller, 2005; Petford et al., 2019) and prioritizing conservation areas, thereby reducing the effort and cost associated with managing endangered species (Wilson et al., 2011).

In the last two decades, advanced modeling strategies, which statistically correlate species' known occurrence records with environmental data are frequently used in ecological studies (Briscoe et al., 2019; Guisan & Thuiller, 2005; Peterson & Soberón, 2012; Rodríguez et al., 2019; Singer et al., 2018). These models predict habitat suitability and species distribution range, generally termed ecological niche models or species distribution models (SDM) (Peterson & Soberón, 2012; Rodríguez et al., 2019). The projected results of SDM can generate answers to questions in ecology, biogeography, evolution, conservation biology, and climate change research (Guisan & Thuiller, 2005).

For example, Petford et al. (2019) investigated the distribution of five understudied, endemic, rupicolous reptiles from the Soutpansberg Mountains in South Africa. Using MaxEnt, a machine learning method that employs maximum entropy to calculate habitat suitability probabilities, they developed SDMs to identify the abiotic variables associated with these species' distributions. Additionally, Wilson et al. (2011) proposed a three‐phase conservation strategy by constructing SDMs and identifying the abiotic variables associated with the habitats suitable for the globally endangered bivalve *Margaritifera margaritifera*.

In this paper, we constructed a species distribution model to map the remaining suitable habitat of the endemic and critically endangered *A. beershebensis*. Due to its small, endemic, and highly fragmented distribution, in addition to its habitat being degraded and altered (Werner & Disi, 2006), it is essential to increase our scientific knowledge about *A. beershebensis* to help focus management and conservation efforts. The constructed SDM model allowed us to quantify the habitat requirements of this critically endangered species, reflecting its ecological niche, and to identify the remaining suitable areas it can inhabit, provided these that they are not subjected to further anthropogenic changes and destruction.

## 2 METHODS

### 2.1 Study area

**FIGURE 1:**
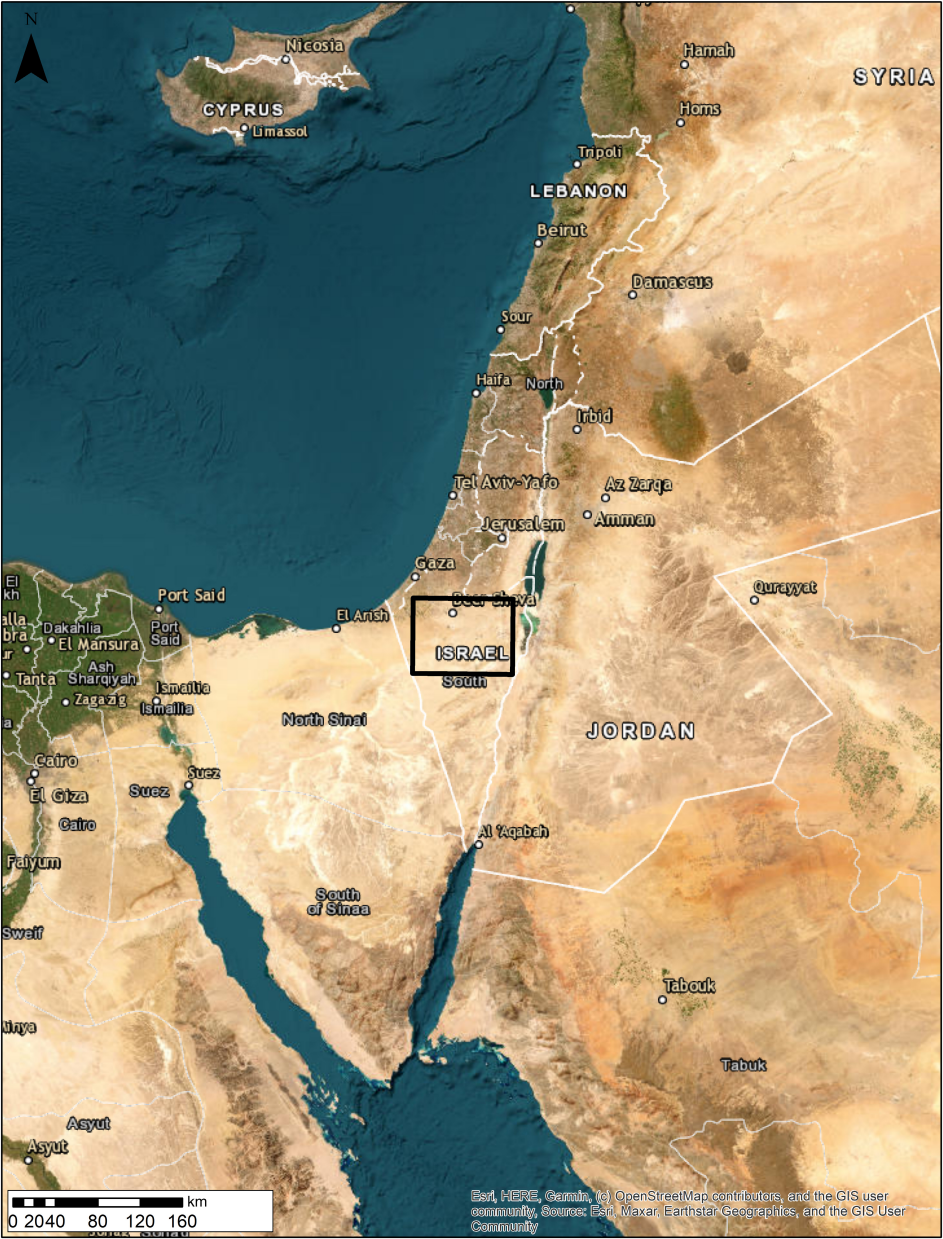
Black square shows the modeled study area, the historical range of *Acanthodactylus beershebensi*s, across the Negev Desert and southern Judea Desert of Israel.

There is limited information regarding the precise historical range of *A. beershebensis*. However, previous studies described this species distribution as endemic to the loess plains across the Negev Desert and southern Judea Desert of Israel. Specifically, the historical range of this species was described as extending from Ovdat (30.794584 and 34.774054) in the south to Beit‐Kama (31.447625 and 34.761953) in the north, and from Arad (31.257105 and 35.215651) in the east to HaBsor (31.282461 and 34.449675) in the west (Bouskila, 2002; Shacham, 2009). We, therefore, defined the study area according to this historical range distribution. We assumed that due to the reported habitat loss (Bouskila, 2002; Hawlena et al., 2010; Shacham, 2009), the remaining suitable habitats are within the limits of the reported historical range. Using ArcMap 10.8.2, we created a rectangular polygon according to these boundaries (Figure 1; bottom left = 30.4973032091726, 34.2693018478088; top right = 31.5383331911955, 35.476584063109), serving as the entire potential distribution area.

### 2.2 Study species

Similarly to all members of the *Acanthodactylus* genus, the Be'er Sheva fringe‐fingered lizard inhabits semi‐arid and arid ecosystems (Tamar et al., 2016). It is a ground‐dwelling insectivorous diurnal lizard that actively forages for small insects, specializing in eating ants (Family: Formicidae) and termites (Infraorder: Isoptera) (Bouskila & Amitai, 2003; Goldberg, 2014; Hawlena & Pérez‐Mellado, 2009). This species quickly escapes into vegetation or a burrow when spotting a potential threat. The individual home range size is approximately 600 m^2^ (Hawlena & Pérez‐Mellado, 2009), and it contains several escape burrows constructed by the lizard (Tesler I. personal observations). Gravid females dig burrows to lay their eggs, which are laid and buried in the loess soil and then naturally incubated (Bar & Haimovitch 2011; Bouskila & Amitai, 2003).

As an ectothermic diurnal reptile, *A. beershebensis* is adapted to the desert environment it inhabits. On hot days, individuals bask before activity in the morning and the afternoon. On cold, windy, and rainy days, when conditions are unfavorable for the lizards' activity, they remain in their burrows (Tesler I. personal observations).

*Acanthodactylus beershebensis* is considered a conservation priority reptile species in Israel, inhabiting one of the world's biodiversity hotspots (Carretero et al., 2011). Werner (2016) wrote that “it is the only species of reptile known to be endemic to the borders of the state of Israel so that the responsibility for its existence devolves on us (Israelis) alone.” Within this species' small and fragmented distribution, certain areas may be protected either directly by stated nature reserves or national parks or indirectly by being declared as closed military firing zones, which in some cases may limit anthropogenic disturbances.

### 2.3 Occurrence data

Occurrence records were collected from four different data sources: (1) the Israeli Nature and Parks Authority (INPA; *n* = 345); (2) the Zoological Museum of Tel Aviv University (*n* = 62); (3) Hamaarag Institute (*n* = 30); and (4) personal/our field observations (*n* = 68), resulting in 505 observations. Experienced researchers and workers collected these presence‐only data as part of field surveys and during random encounters in the field. All observations used in the models were collected between 2014 and 2022.

Using ArcMap 10.8.2, an average nearest neighbor test showed observations to be clustered (*z*‐score = −32.09) and potentially biased. Similarly, a kernel density analysis also showed observations clustered in five areas. Due to this spatial clustering, an analysis was performed to obtain an observation dataset with a random distribution and no spatial biases. Observations were clipped with an increasing tolerance radius, beginning with a radius of the average distance between two points, 365 m. An average nearest neighbor test was performed for the different clipping radiuses (Guisan & Thuiller, 2005; Mathur et al., 2023; Zurell et al., 2020). This optimization analysis showed that clipping the observation data with a tolerance radius of 1000 m generated a random pattern (*z*‐score = −1.29), generating 59 distinct data points representing the 505 field observations.

### 2.4 Environmental data

*Acanthodactylus beershebensis* is an endemic species with a narrow geographical range, a specialized ecological niche, small and declining population sizes, and a sedentary nature with low dispersal rates (See Section 1: Introduction). Based on its known life‐history traits and reported behavior, we predicted that the following variables are likely to affect the species' habitat suitability: (1) temperature, sunlight, and precipitation for thermoregulation and prey presence; (2) ground substrate and slope for digging burrows, movement, and foraging; and (3) vegetation cover for shelter. Additionally, anthropogenic development has been shown to negatively affect this species presence (Hawlena & Bouskila, 2006; Hawlena et al., 2010) leading to habitat fragmentation. Therefore, the species is expected to prefer hot and dry climatic conditions in moderate slopes with mild vegetation cover and loess soils. We posited that topographic, climatic, habitat, and edaphic variables would be associated with the species' spatial distribution (Table 1). The following data layers were used in the SDM (Table 1): elevation, slope, aspect, annual mean rainfall, land cover, soil groups, vegetation cover, and natural area continuity. Additionally, the “Worldclim 2.1” dataset (worldclim.org), published by the United Nations Climate Technology Centre and Network, was accessed to download 19 bioclimatic variables (Fick & Hijmans, 2017). These variables are derived from the monthly temperature and rainfall values and are the average for the years 1970–2000. Categorical variables (i.e., land cover and soil groups) were represented numerically to assess their effect on habitat suitability.

**TABLE 1:** Abiotic data layers predicted to explain the distribution of *Acanthodactylus beershebensis*, their resolution, source, expected correlation, and whether they were included as explanatory variables in the final species distribution model.

Abiotic data layer	Resolution	Source	Predicted association	Included in the final model
Terrain: Ellipsoidal height	0.25 × 0.25 m	Esri ArcGIS Online Data	Negative correlation	No—removed due to low contribution
Terrain: Slope in degrees	0.25 × 0.25 m	Esri ArcGIS Online Data	Negative correlation	Yes
Terrain: Aspect	0.25 × 0.25 m	Esri ArcGIS Online Data	Preference for south‐facing slopes	Yes
Esri 2020 land cover	10 × 10 m	Esri ArcGIS Online Data	Preference for bare land	Yes
Vegetation cover	10 × 10 m	Hamaarag Institute	Negative correlation	Yes
DMT land cover	25 × 25 m	Hamaarag Institute	Preference for bare land	Yes
Digital elevation model (DEM)	25 × 25 m	Hamaarag Institute	Negative correlation	No—removed due to multicollinearity
Natural area continuity (Rezef full)	25 × 25 m	Hamaarag Institute	Positive correlation	Yes
Annual mean rainfall	250 × 250 m	Hamaarag Institute	Negative correlation	Yes
Soil groups	1:50,000	KKL‐JNF	Preference for loess soil	Yes
BIO1—Annual mean temperature	1000 × 1000 m	Worldclim 2.1	Quadratic correlation	No—removed due to multicollinearity
BIO2—Mean diurnal temperature range	1000 × 1000 m	Worldclim 2.1	Quadratic correlation	Yes
BIO3—Isothermality (BIO2/BIO7 × 100)	1000 × 1000 m	Worldclim 2.1	Quadratic correlation	No—removed due to low contribution
BIO4—Temperature seasonality (SD × 100)	1000 × 1000 m	Worldclim 2.1	Quadratic correlation	No—removed due to multicollinearity
BIO5—Maximum temperature of warmest month	1000 × 1000 m	Worldclim 2.1	Quadratic correlation	No—removed due to low contribution
BIO6—Minimum temperature of coldest month	1000 × 1000 m	Worldclim 2.1	Negative correlation	No—removed due to multicollinearity
BIO7—Temperature annual range	1000 × 1000 m	Worldclim 2.1	Quadratic correlation	Yes
BIO8—Mean temperature of wettest quarter	1000 × 1000 m	Worldclim 2.1	No correlation	No—removed due to multicollinearity
BIO9—Mean temperature of driest quarter	1000 × 1000 m	Worldclim 2.1	No correlation	No—removed due to multicollinearity
BIO10—Mean temperature of warmest quarter	1000 × 1000 m	Worldclim 2.1	No correlation	No—removed due to multicollinearity
BIO11—Mean temperature of coldest quarter	1000 × 1000 m	Worldclim 2.1	Negative correlation	No—removed due to multicollinearity
BIO12—Annual precipitation	1000 × 1000 m	Worldclim 2.1	negative correlation	No—removed due to low contribution
BIO13—Precipitation of wettest month	1000 × 1000 m	Worldclim 2.1	Negative correlation	No—removed due to multicollinearity
BIO14 —Precipitation of driest month	1000 × 1000 m	Worldclim 2.1	Negative correlation	No—removed due to multicollinearity
BIO15—Precipitation seasonality (coefficient of variation)	1000 × 1000 m	Worldclim 2.1	Negative correlation	No—removed due to multicollinearity
BIO16—Precipitation of wettest quarter	1000 × 1000 m	Worldclim 2.1	Negative correlation	No—removed due to multicollinearity
BIO17—Precipitation of driest quarter	1000 × 1000 m	Worldclim 2.1	Negative correlation	No—removed due to multicollinearity
BIO18—Precipitation of warmest quarter	1000 × 1000 m	Worldclim 2.1	Negative correlation	No—removed due to multicollinearity
BIO19—Precipitation of coldest quarter	1000 × 1000 m	Worldclim 2.1	Negative correlation	No—removed due to multicollinearity

All the environmental data layers were cut to fit the geographic extent of the study area at a pixel size of 10 × 10 m. Cell values were projected using ArcMap's “Build Pyramids” tool to represent the data in a reduced resolution using the nearest neighbor resampling method which used the value of the closest cell to assign a value to the output cell. The correlation among layers was investigated by generating Pearson's correlation coefficient matrices using the Band Collections Statistics Tool. The final species distribution model included the following environmental data layers, which presented correlation coefficients below 0.85; elevation, slope, aspect, annual mean rainfall, ESRI land cover, DMT land cover, soil groups, vegetation cover, natural area continuity, BIO2—mean diurnal temperature range, BIO3—isothermality, BIO5–maximum temperature of warmest month, BIO7—temperature annual range, and BIO12 —annual precipitation. Clipped layers were then converted to ASCII format for use in MaxEnt. After gaining preliminary model results, the following redundant variables that contributed <1% to the model fit were removed; elevation, BIO3—isothermality, BIO5—maximum temperature of the warmest month, and BIO12—annual precipitation.

### 2.5 Species distribution modeling with MaxEnt

MaxEnt estimates a target probability distribution by calculating the maximum entropy probability distribution, making it applicable to species distribution modeling (Mathur et al., 2023). As a presence‐only model, MaxEnt compares the distribution of presence records along environmental gradients to the distribution of background points drawn randomly from the study area using a background sample (Mathur et al., 2023). MaxEnt software version 3.4.4 was used to model the habitat suitability of the endemic and critically endangered *A. beershebensis* by combining the prepared environmental layers and species' occurrence records. Species distribution modeling was conducted following the steps and ODMAP protocol suggested by Zurell et al. (2020) (Appendix S1).

As accepted, 75% of the data were set as training data, and 25% were used as test data (Elith et al., 2006). Other test parameters were left as default. The number of randomly generated background points was set to 10,000. Linear, quadratic, and hinge properties were employed. After the first run of the model, variables that did not contribute to the model fit and were redundant (<1% contribution) were removed, and the model was rerun. The layers used in the final model are noted in Table 1.

Threshold‐independent receiver operating characteristic (ROC) analyses were used to calibrate and validate the robustness of the MaxEnt model evaluation. The accuracy of the model predictions was evaluated using the area under the receiver operating curve (AUC). Additionally, MaxEnt outputs a Jackknife test of variable importance in which each variable is excluded in turn, and a model is created with the remaining variables.

### 2.6 Distribution map analysis

The results of the species distribution model were further investigated to identify the areas of likely distribution and suitable habitats still available for *A. beershebensis*. To plan effective conservation strategies and generate applicable knowledge, the continuous habitat suitability output of the model was converted to binary data of suitable and unsuitable habitats. Habitats were defined based on a threshold value generated by MaxEnt, using the method that maximizes the sum of sensitivity and specificity. This method has been shown to be a promising threshold selection method when only presence data are available (Liu et al., 2013).

Four data layers of human developments were downloaded from the Esri ArcGIS online server: (1) World Roads—major roads, highways, local roads, and world ferries. (2) “Built Area”—clipped from Sentinel‐2 10 m land cover time series of the world. Produced by Impact Observatory, Microsoft, and Esri. (3) Public Agricultural Plots and (4) Pasture Parcels produced by the Israeli Ministry of Agriculture.

To correct for temporal and spatial discrepancy of the 59 averaged observations, areas of suitable habitats that overlapped with the human development layers were erased and considered unsuitable for the study species. The remaining suitable habitats were further examined. Habitats can be directly protected when residing within nature reserves or national parks or indirectly by being declared as closed military firing zones. Therefore, the “Firing Zone” and “Reservations and Parks INPA” data layers, both produced by the INPA, were downloaded from the Esri ArcGIS online server. Using the “Calculate Geometry” function in ArcMap 10.8.2, the total area of suitable habitat within the historical range of *A. beershebensis* was calculated for the continuous and binary model outputs. Additionally, the total area of protected suitable habitats was calculated.

## 3 RESULTS

**FIGURE 2:**
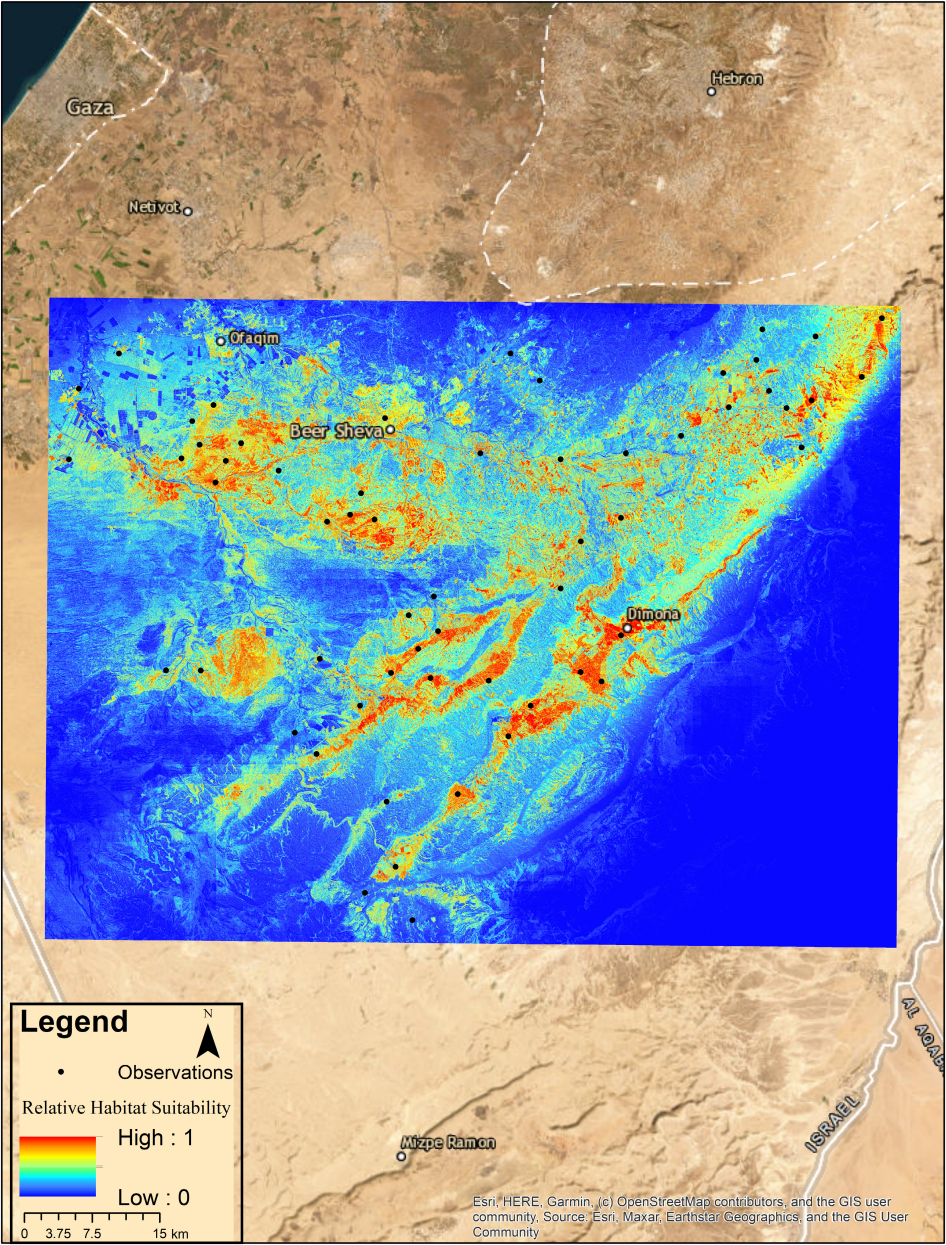
Species distribution model for *Acanthodactylus beershebensis* across the species historical range, based on 505 observations scaled down to 59 distinct spatially random data points (black circles). Pixels of 10 × 10 m show areas of low suitability in blue and high suitability in red.

**FIGURE 3:**
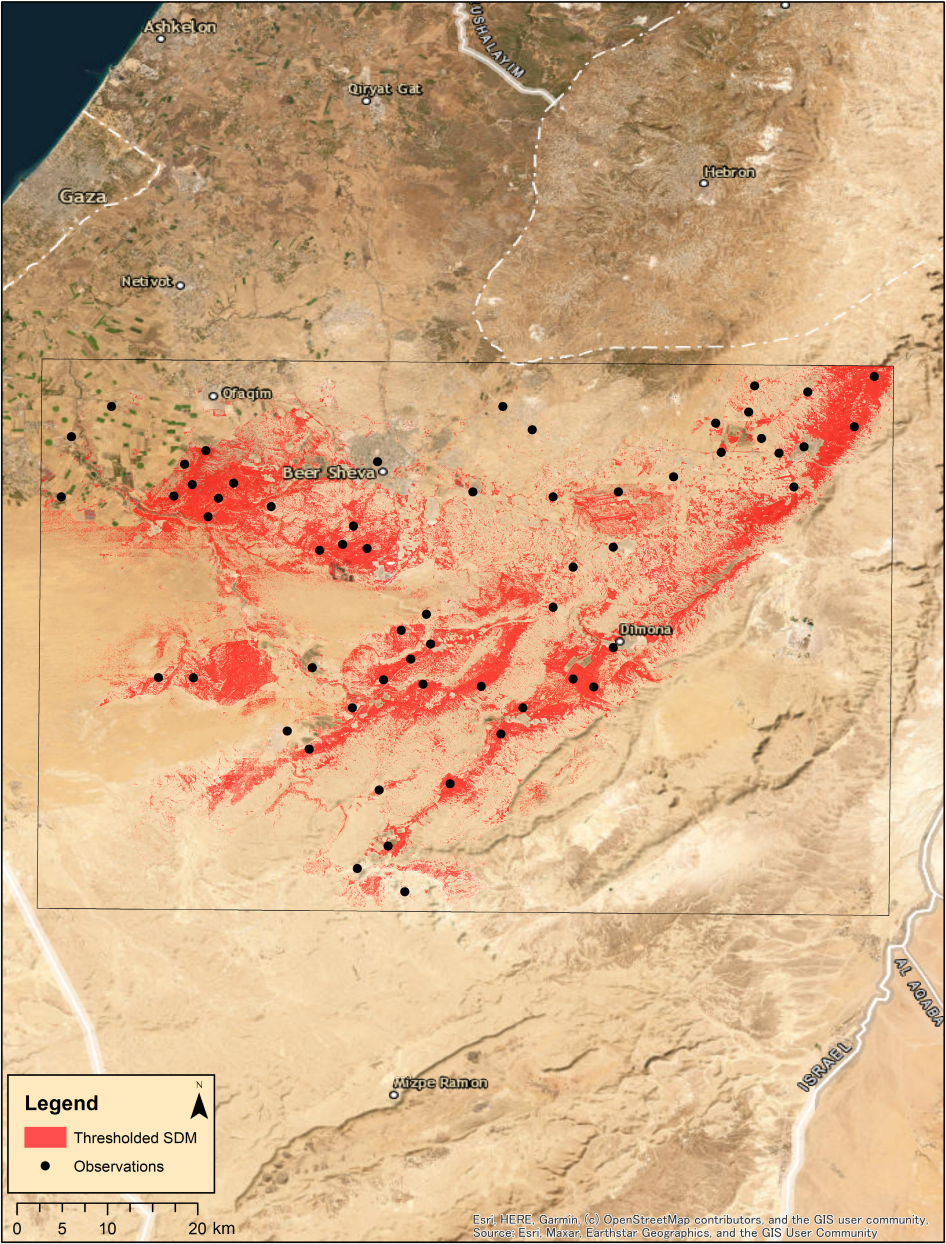
Binary habitat suitability map generated using the results of the species distribution model for *Acanthodactylus beershebensis* across the species historical range, based on 505 observations scaled down to 59 distinct spatially random data points (black circles). The threshold was set as 0.46. Pixels of 10 × 10 m show areas of suitable habitats in red.

The SDM performed well, with an AUC value of 0.89. Model results produced two habitat suitability maps with a pixel size of 10 × 10 m. Every pixel is assigned a relative habitat suitability value based on the model variables. The first map presents continuous relative habitat suitability values ranging from 0 (unsuitable habitat) to 1 (suitable habitat; Figure 2) and the second with binary data (0 or 1), based on a threshold value of 0.46 as generated by maximizing the sum of sensitivity and specificity method (Figure 3).

Environmental variables that contributed most to the MaxEnt model were annual mean rainfall (48.90%), terrain slope (17.80%), and BIO2 (mean diurnal range; 9.80%). Results of the Jackknife analysis showed that the variable with the highest contribution to the training gain of the model, when used separately, is annual mean rainfall. Therefore, it appears to be the most useful to the model, and when omitted, it decreased the gain the most. Additionally, plots from the Jackknife analysis of AUC values show that Natural Area Continuity (“Rezef Full”) and BIO7 (Temperature annual range) presented the highest AUC values when used separately.

Response curves produced by the model present the habitat requirements of *A. beershebensis* (Appendix S2). As expected from the species' historical range, habitat suitability was highest in areas with approximately 60–250 mm of annual rainfall characterized by semi‐arid Negev Scrublands. Furthermore, habitat suitability was higher within an annual temperature range (BIO7) of 25°C–27.5°C. The response curves produced by the model also illustrated that the species' suitable habitat was negatively correlated with slope (being highest in a 0° slope) and vegetation cover (percent of the pixel covered by vegetation). However, habitat suitability was higher in bare loess soil and north‐facing aspects.

**FIGURE 4:**
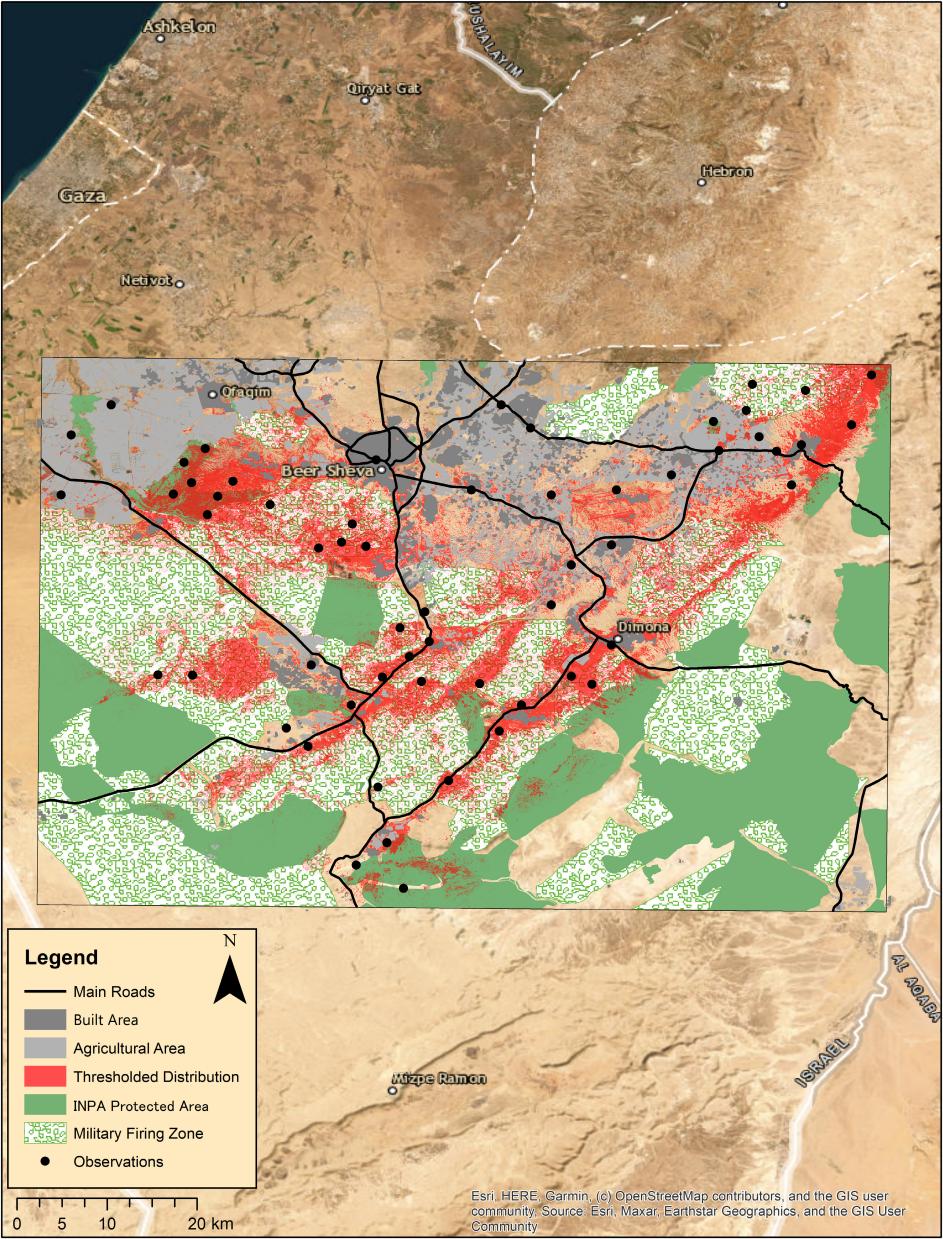
Remaining suitable habitat for *Acanthodactylus beershebensis* based on the SDM. Built areas are dark gray, agricultural areas are light gray, INPA‐protected areas are green, military firing zones are white‐green, and main roads are in black lines.

The model showed that within the species' historical range, the size of its potentially suitable habitat is 1350.73 km^2^ and that sites of high habitat suitability are adjacent to roads, agriculture areas, and built areas (Figure 4). These anthropogenic effects decrease the size of the remaining suitable habitat. Therefore, areas of human development (main roads, built areas, agricultural plots, and pasture parcels) were erased and considered unsuitable for the study species. The size of the remaining suitable habitat throughout the historic range was 995.04 km^2^ (Figure 4). The remaining suitable habitat remains mostly unprotected and under possible further adverse anthropogenic effects, with only 91.72 km^2^ (~9%) within nature reserves and parks protected by the Israel Nature and Parks Authority. An additional 587.11 km^2^ of the suitable habitat (~59%) is within declared military firing zones and thus may be considered partially and indirectly protected.

## 4 DISCUSSION

Endemic species are typically more susceptible to habitat changes, manifested in accelerated population declines (Díaz et al., 2019; IPBES, 2019) and higher extinction rates (Işik, 2011). Using SDM to generate relative habitat suitability maps and identify species' potential and current distribution provides the basic knowledge needed to plan effective conservation strategies (Guisan & Thuiller, 2005; Petford et al., 2019) and to prioritize conservation areas (Wilson et al., 2011).

This study is the first attempt to map the remaining suitable habitat of the endemic and critically endangered lizard *A. beershebensis* based on the results of a species distribution model. The generated distribution maps suggest that the species' suitable habitat of the species may exceed the modeled area to the northeast. However, we assume this area to be relatively small and confined due to changes in climatic conditions and to the steep canyons and wadis that lead to the Dead Sea, thus decreasing habitat suitability and potential distribution.

The model quantified the species' habitat requirements and illustrated the attributes that dictate its narrow geographical range and specialized ecological niche, confirming previous expert knowledge (Bar & Haimovitch 2011; Bouskila & Amitai, 2003; Shacham, 2009; Werner, 2016). As a terrestrial species, *A. beershebensis* relies on the habitat's attributes for different activity types (e.g., foraging, thermoregulation, escaping from predators, burrowing, and reproduction/egg deposition) and hence for maximizing its fitness. Activity in non‐preferred habitats can negatively affect fitness by influencing, among others, locomotion, diet, thermoregulation, reproduction, and escape from predators (Goodman, 2007).

The model indicated that *A. beershebensis* suitable habitats are associated with arid loess plains characterized by scattered and low vegetation cover, located mainly on north‐facing aspects. As expected, the suitable habitat of this desert‐adapted species is defined by arid to semi‐arid climatic conditions, consistent with Tamar et al. (2016), reporting that the *Acanthodactylus* is an arid‐adapted genus. Lizard species of this genus are commonly known as fringe‐fingered lizards due to their distinctive scalation—jagged scales along the fingers, which are especially noticeable on the hind legs where the fingers are longer (Shacham, 2009; Tamar et al., 2016). These jagged scales increase the surface area of the lizards' fingers to improve traction and grip on light sandy soils (Savvides et al., 2019). Shacham (2009) suggested a correlation between soil compression and the relative size of the jagged scales and fingers of different *Acanthodactylus* species. Species more adapted to loosen, mobile sandy soils (e.g., shifting dunes) possess relatively more prominent and distinguished scales. Adapted to loess soils, which are relatively compact and stable, *A. beershebensis* has smaller and less distinguished jagged scales than all other five *Acanthodactylus* species found in Israel (Shacham, 2009).

*Acanthodactylus beershebensis* is a habitat specialist adapted to loess soils. Its preferred habitat attributes align with the behavior of this morphologically distinct species (Moravec et al., 1999). Due to its habitat preferences, the morphological traits of *A. beershebensis* are likely dictated by its habitat attributes. Similarly, Goodman et al. (2008) showed a shift in morphology and performance in response to the increased use of rocky habitats of lygosomine skinks. The physical attributes of the habitat increased selection for morphological adaptations that increase the species' fitness in this group of lizards. Irschick and Losos (1999) showed that the sprinting performance of *Anolis* lizard species varied across different substrate diameters and that lizards tended to avoid surfaces that impaired their maximal performance. Li et al. (2011) showed that grain size significantly affected the running speed of the toad‐headed lizard (*Phrynocephalus frontalis*). Additionally, Hibbitts et al. (2013) showed that terrain slope, soil type, and ground cover affected the microhabitat selection of two lizard species (*Sceloporus arenicolus* and *Uta stansburiana*).

Previous studies have identified the preferred habitat and substrate of similar species. Zaady and Bouskila (2002) showed that *A. boskianus* and *A. opheodurus* prefer to burrow on temporary soil crusts while avoiding all other soil surfaces. Shenbrot and Krasnov (1997) found that the density of the desert lizard species *A. boskianus* and *Mesalina bahaeldini* (as *M. guttulata*) is associated with soil structure and that of *M. olivieri* with vegetation structure. Notably, in contrast to the SDM approach, in previous studies, habitat preferences of lizard species were inferred by calculating the proportion of time they utilized different habitat types (e.g., Agarwal et al., 2015; Pérez‐Mellado, 1992; Shenbrot & Krasnov, 1997) which requires considerable effort and time.

Species distribution modeling is essential for illustrating lizard habitat requirements and identifying the habitats' attributes associated with their spatial distribution. SDMs have been shown to accurately predict current and future species distributions (Mousikos et al., 2021) and have been used successfully to model lizard species' distributions. For instance, Petrosyan et al. (2019) modeled the distribution of three lizard species of the genus *Darevskia*. Thonis and Lister (2019) used species distribution models to show that the suitable habitat for 10 species of *Anolis* lizards is predicted to decrease in the future.

The model can explain the species' physical boundaries, limiting its fundamental niche. To the southeast of the modeled area, the species' distribution seems limited by the steep slopes of Hatira Ridge. In the southwest, the species is limited by changes in soil substrate. Finally, the species is limited to the north due to different climate conditions. Within this narrow and limited geographical range and based on the calculated suitability threshold, an area of 995.04 km^2^ experiencing a low level of anthropogenic disturbances seems suitable for *A. beershebensis*. As presented in the results, only 9% of the remaining suitable habitat is within protected nature reserves and parks. The majority of the remaining suitable habitat, 59%, is within declared military firing zones and thus may be considered indirectly protected. Such areas are closed to the public, uninhabited, and undeveloped. However, it is essential to note that some suitable areas within the military firing zones are degraded by military activity, such as building construction, heavy vehicle traffic, or live ammunition training.

This study can assist in further research and conservation of this endemic and critically endangered species. Ecologists should aim future surveys to areas of suitable habitat to examine the species' demographic rates and identify possible source and sink populations. Furthermore, the model results enable conservationists and decision‐makers to locate areas of critical suitable habitat and show where future habitat degradation or destruction should be prevented. Increasing the number of suitable habitats that are protected by declaring more nature reserves can assist in conserving *A. beershebensis* and other species occurring within its geographical distribution.

Indeed, previous studies showed that planting trees may lead to species replacement and even local extinction of *A. beershebensis* (Hawlena & Bouskila, 2006; Hawlena et al., 2010). Additionally, Pafilis et al. (2013) showed that grazing has a negative effect on the occurrence of the insular lizard (*Podarcis gaigeae*) by impacting insect populations, thus decreasing food availability for lizards. Anthropogenic alterations to the habitat negatively affect biodiversity and species fitness. Consequently, habitat protection increases reptile species richness (Attum et al., 2006). Furthermore, Attum et al. (2006) reported that *A. longipes* is more abundant in fenced areas protected from grazing and agriculture.

Our study provides the first attempt to map the remaining suitable habitat of the endemic and critically endangered *A. beershebensis* based on the results of a species distribution model. This study is the first to quantify the species' habitat requirements while illustrating its narrow geographical range and specialized ecological niche. Finally, the model results can be used to design conservation strategies and plans to protect the remaining suitable habitats for *A. beershebensis* and to support the establishment of large nature reserves to protect the lizard and other loess scrublands threatened species such as McQueen's Bustard (*Chlamydotis macqueenii*) and Buxton's Jird (*Meriones sacramenti*).

## AUTHOR CONTRIBUTIONS

**Sefi J. A. Horesh:** Conceptualization (equal); data curation (lead); formal analysis (lead); methodology (lead); software (lead); writing – original draft (equal); writing – review and editing (supporting). **Ofer Ovadia:** Conceptualization (equal); data curation (supporting); methodology (supporting); supervision (lead); writing – original draft (equal); writing – review and editing (lead).

## CONFLICT OF INTEREST STATEMENT

The authors declare no conflict of interest.

### OPEN RESEARCH BADGES

This article has earned Open Data and Open Materials badges. Data and materials are available at https://doi.org/10.5061/dryad.pnvx0k6vp.

## Supporting information


Appendix S1.

Appendix S2.

## Data Availability

All data used to create the model is available online. Dryad Reviewer Sharing Link: https://datadryad.org/stash/share/_DlTQIC5zUgpsjQlB‐x_8A9‐U0E9Fm9IK1my6RQvWyk. Observation records of *Acanthodactylus beershebensis* used in the model and the study area polygon were uploaded to Dryad: https://doi.org/10.5061/dryad.pnvx0k6vp. All environmental layers used in the model were downloaded freely from Esri ArcGIS Online Data and from the Worldclim website: https://worldclim.org/data/worldclim21.html. Species distribution model ODMAP protocol, model results and supplemental information were uploaded to Dryad: https://doi.org/10.5061/dryad.pnvx0k6vp.
