# Supplementary material for: Predicting habitat suitability of the critically endangered Be'er Sheva fringe‐fingered lizard
Source: Ecol Evol. 2024 Aug 16;14(8):e70108. doi: 10.1002/ece3.70108 (PMC11327611; doi:10.1002/ece3.70108)
Supplement: Supplementary file 1 — Appendix S1. [file ECE3-14-e70108-s001.docx]

Predicting Habitat Suitability of the Critically Endangered Beer Sheva Fringe-fingered Lizard

– ODMAP Protocol –

Sefi JA Horesh, Ofer Ovadia

2023-10-25

## Overview

#### Authorship

Contact: [adizesho@post.bgu.ac.il](mailto:adizesho@post.bgu.ac.il)

Study link: NA

#### Model objective

Model objective: Mapping and interpolation

Target output: Suitable habitat

#### Focal Taxon

Focal Taxon: *Acanthodactylus beershebensis*

#### Location

Location: Negev Desert, Israel

#### Scale of Analysis

Spatial extent: 30.4973032091726, 31.5383331911955, 34.2693018478088, 35.476584063109 (xmin, xmax, ymin, ymax)

Spatial resolution: 0.01

Temporal extent: 2014 - 2022

Temporal resolution: NA

Boundary: rectangle

#### Biodiversity data

Observation type: field survey

Response data type: point occurrence

#### Predictors

Predictor types: climatic, topographic, edaphic, habitat

#### Hypotheses

Hypotheses: Current data shows that the species’ activity and life history traits depend on temperature, sunlight, precipitation, ground substrate, vegetation cover, and anthropogenic development. The species is expected to prefer hot and dry climatic conditions and loess soils with moderate slopes and low vegetation cover.

#### Assumptions

Model assumptions: 1) Independence of species observations - each species record represents new information. 2) All relevant environmental drivers are included in the model. 3) Predictors are free of error

#### Algorithms

Modelling techniques: maxent

Model complexity: Topographic, climatic, habitat, and edaphic variables seem to be associated with the species’ spatial distribution; therefore, the model needed to incorporate numerous environmental data layers.

Model averaging: NA

#### Workflow

Model workflow: 1) Occurrence data accumulation, spatial bias analyses, and correction. 2) Preparation of relevant environmental data layers. 3) Analysis for correlation among environmental data layers. 4) Species distribution modeling with MaxEnt. 5) Analysis of final model results and distribution map.

#### Software

Software: Maximum Entropy (MaxEnt) Species Distribution Modeling, Version 3.4.4

Code availability: NA

Data availability: NA

## Data

#### Biodiversity data

Taxon names: Acanthodactylus beershebensis

Taxonomic reference system: Morvec et al. 1999

Ecological level: species

Data sources: (1) Israel Nature and Parks Authority; 2010-2022; *Acanthodactylus beershebensis* observations; Data – Scientific Data Department, Israel Nature and Parks Authority. (2) Hamaarag – Israel’s National Ecosystem Assessment Program; 2010-2022; *Acanthodactylus beershebensis* observations; The Steinhardt Museum of Natural History, Tel Aviv University. (3) Shai Meiri’s Lab; 2014-2021; *Acanthodactylus beershebensis* observations; The Steinhardt Museum of Natural History, Tel Aviv University. (4) Sefi Horesh; 2020-2022; *Acanthodactylus beershebensis* observations.

Sampling design: NA

Sample size: 505

Clipping: Negev desert, Israel

Scaling: To correct for spatial bias, the observation data was clipped with a tolerance radius of 1000 meters and generated a random pattern, resulting in 59 distinct data points representing the 505 field observations.

Cleaning: NA

Absence data: NA

Background data: Ten thousand background points were drawn randomly from the study area.

Errors and biases: NA

#### Data partitioning

Training data: 75 percent

Validation data: NA

Test data: 25 precent

#### Predictor variables

Predictor variables: Elevation, slope, aspect, annual mean rainfall, land cover, soil groups, vegetation cover, natural area continuity, and 19 worldclim bioclimatic variables.

Data sources: (1) Esri ArcGIS Online Data (2) Hamaarag Institute (3) Worldclim 2.1

Spatial extent: 30.4973032091726, 31.5383331911955, 34.2693018478088, 35.476584063109 (xmin, xmax, ymin, ymax)

Spatial resolution: Varied

Coordinate reference system: WSG 1984

Temporal extent: Varied

Temporal resolution: NA

Data processing: All the environmental data layers were cut to fit the geographic extent of the study area at a pixel size of 10x10 meters. Cell values were projected using ArcMap 10.8.2 “Convert Units” cell size projection method.

Errors and biases: NA

Dimension reduction: NA

#### Transfer data

Data sources: NA

Spatial extent: NA, NA, NA, NA (xmin, xmax, ymin, ymax)

Spatial resolution: NA

Temporal extent: NA

Temporal resolution: NA

Models and scenarios: NA

Data processing: NA

Quantification of Novelty: NA

## Model

#### Variable pre-selection

Variable pre-selection: Based on previously published data and expert knowledge.

#### Multicollinearity

Multicollinearity: The correlation among layers was investigated by generating Pearson’s Correlation Coefficient matrices using the Band Collections Statistics Tool in ArcMap 10.8.2. The final species distribution model included only layers with a correlation coefficient below 0.85.

#### Model settings

MaxEnt: FeatureSet (Linear, Quadratic, Product, Threshold, Hinge, Categorical), FeatureRule (Auto Feature), RegularizationMultiplierSet (1), RegularizationRule (Multiply all automatic regularization parameters by this number), convergenceThresholdSet (0.00001)

Model settings (extrapolation): NA

#### Model estimates

Coefficients: Permutations

Parameter uncertainty: NA

Variable importance: Jackknife analyses and response curves

#### Model selection - model averaging - ensembles

Model selection: NA

Model averaging: NA

Model ensembles: NA

#### Analysis and Correction of non-independence

Spatial autocorrelation: Observations were clipped with a tolerance radius of 1000 meters and generated a random pattern.

Temporal autocorrelation: NA

Nested data: NA

#### Threshold selection

Threshold selection: Maximize the sum of sensitivity and specificity method

## Assessment

#### Performance statistics

Performance on training data: AUC, ROC

Performance on validation data: NA

Performance on test data: AUC, ROC

#### Plausibility check

Response shapes: Response curves

Expert judgment: Map display

## Prediction

#### Prediction output

Prediction unit: Continuous occurrence probabilities and potential presence derived by thresholding.

Post-processing: Clipped areas of anthropogenic presence using the threshold results.

#### Uncertainty quantification

Algorithmic uncertainty: NA

Input data uncertainty: NA

Parameter uncertainty: NA

Scenario uncertainty: NA

Novel environments: Masking and counting the amount of protected suitable habitat.
